# Dendritic diameters affect the spatial variability of intracellular calcium dynamics in computer models

**DOI:** 10.3389/fncel.2014.00168

**Published:** 2014-07-23

**Authors:** Haroon Anwar, Christopher J. Roome, Hermina Nedelescu, Weiliang Chen, Bernd Kuhn, Erik De Schutter

**Affiliations:** ^1^Theoretical Neurobiology and Neuroengineering, University of AntwerpWilrijk, Belgium; ^2^Computational Neuroscience Unit, Okinawa Institute of Science and TechnologyOnna-Son, Okinawa, Japan; ^3^Optical Neuroimaging Unit, Okinawa Institute of Science and TechnologyOnna-Son, Okinawa, Japan

**Keywords:** intracellular calcium, calcium concentration, calcium buffering, diffusion, dendritic diameter, compartmentalization, active dendrite, morphology

## Abstract

There is growing interest in understanding calcium dynamics in dendrites, both experimentally and computationally. Many processes influence these dynamics, but in dendrites there is a strong contribution of morphology because the peak calcium levels are strongly determined by the surface to volume ratio (SVR) of each branch, which is inversely related to branch diameter. In this study we explore the predicted variance of dendritic calcium concentrations due to local changes in dendrite diameter and how this is affected by the modeling approach used. We investigate this in a model of dendritic calcium spiking in different reconstructions of cerebellar Purkinje cells and in morphological analysis of neocortical and hippocampal pyramidal neurons. We report that many published models neglect diameter-dependent effects on calcium concentration and show how to implement this correctly in the NEURON simulator, both for phenomenological pool based models and for implementations using radial 1D diffusion. More detailed modeling requires simulation of 3D diffusion and we demonstrate that this does not dissipate the local concentration variance due to changes of dendritic diameter. In many cases 1D diffusion of models of calcium buffering give a good approximation provided an increased morphological resolution is implemented.

## Introduction

Intracellular Ca^2+^ has a central role in the information processing capabilities of neuronal dendrites. Ca^2+^ entering through voltage-gated Ca^2+^ channels (VGCC) and ligand-gated channels gives rise to cytosolic Ca^2+^, which in turn controls Ca^2+^-activated K^+^ (K_Ca_) channels during dendritic Ca^2+^ spikes (Goldberg et al., 2004; Womack and Khodakhah, 2004; Kampa and Stuart, 2006). Free cytosolic Ca^2+^ can also activate complex molecular signaling pathways involved in different forms of synaptic and dendritic plasticity (Konnerth et al., 1992; Kampa et al., 2006; Rancz and Hausser, 2006; Canepari and Vogt, 2008; Antunes and De Schutter, 2012). The cytosolic spread and dynamics of Ca^2+^ in dendritic morphologies are controlled by intracellular Ca^2+^ mechanisms like diffusion, endogenous buffers, internal stores, exchangers and pumps (Berridge, 1998; Augustine et al., 2003; Hartmann and Konnerth, 2005). Therefore, correct representation of Ca^2+^ related mechanisms in complex dendritic structures is crucial in construction of biophysically faithful multi-scale models of dendrites.

In addition to intracellular Ca^2+^ mechanisms and ion channel distributions, dendritic geometry has been shown to greatly affect the spatial variability of Ca^2+^ dynamics (Lev-Ram et al., 1992; Regehr and Tank, 1994; Schiller et al., 1995; Holthoff et al., 2002; Rozsa et al., 2004). The effects of dendritic geometry on Ca^2+^ transients are often quantified in terms of the surface to volume ratio (SVR). This is because Ca^2+^ influx scales with membrane surface while the change in Ca^2+^ concentration due to diffusion and buffering strongly depends on the volume. This results in larger amplitude transients expected in small diameter dendrites because they have a large SVR. Considering each dendritic segment as a cylinder, SVR is inversely proportional to the diameter of the cylinder. Therefore, even in the absence of intracellular Ca^2+^ mechanisms (endogenous buffers, internal Ca^2+^ stores) and diffusion, changes in dendritic diameter across the dendrite will result in spatially variable Ca^2+^ levels. Moreover, because Ca^2+^ buffering and diffusion are also affected by geometry, dendrite diameters can also affect the decay time constants of Ca^2+^ transients (Holthoff et al., 2002). In this paper we characterize this spatial variability in Purkinje cell models and explore implementation issues that affect how well a biophysically detailed dendrite model can capture the spatio-temporal variability of Ca^2+^ dynamics caused by local variation of dendrite diameters.

Traditionally, a Ca^2+^ pool with a single relaxation time constant is used to model intracellular Ca^2+^ dynamics (Destexhe et al., 1994). Such models compute the effects of Ca^2+^ influx accurately but combine all removal systems, including diffusion, into one process with a fixed time constant. They usually represent the Ca^2+^ concentration in a submembrane shell with a fixed depth. Previously, we have shown that these pool based models can not capture the complex dynamics of intracellular Ca^2+^ because they fail to simulate the multiple time scales at which interactions between VGCC and K_Ca_ channels occur (Anwar et al., 2012). Here we extend the comparison of Ca^2+^ pool to complex Ca^2+^ dynamics models to the spatial domain. We will show that many model implementations in the literature do not compute correct volumes for the submembrane shell and that accuracy of morphological reconstruction is a more important concern than the limitations of only modeling 1D radial diffusion. In addition we provide detailed instructions on how to model biophysically realistic Ca^2+^ dynamics in compartmental models of dendrites.

## Materials and methods

### Morphology reconstructions

#### Purkinje cell morphologies

Dendritic morphologies of 10 Purkinje cells (PC 3–12) used in this study were obtained from the NeuroMorpho database (http://neuromorpho.org). An additional Purkinje cell morphology (PC 2) used in this study was provided by Ede Rancz and Michael Häusser, UCL, London, UK. Considering the small sample size of available PC neurons (11 cells) and their large variability in dendritic diameters, we decided to obtain an additional morphology (PC 1) with carefully reconstructed diameters.

#### PC morphology with carefully reconstructed diameters

All procedures for the care of animals were according to the Science Council of Japan Guidelines for Proper Conduct of Animal Experiments, and also the guideline approved by OIST Graduate University Animal Resources Section. A 4-week old mouse was anesthetized with isofluorane and decapitated. The cerebellum was removed from the skull and immediately collected into a vial containing ice cold carbogenated ACSF: NaCl 125 mM, KCl 2.5 mM, NaH_2_PO_4_ 1.2 mM, MgSO_4_ 1.9 mM, Glucose 10 mM, NaHCO_3_ 25 mM, CaCl_2_ 2 mM at 300-305 mOsm. Sagittal slices of 250 μm thickness were cut and placed in a recording chamber with carbogenated ACSF. The glass electrode (4 MOhm) was filled with intracellular solution containing potassium gluconate 140 mM, NaCl 10 mM, HEPES 10 mM, EGTA 0.2 mM, MgATP 4 mM, NaGTP 0.4 mM, Phosphocreatine 10 mM and 50 μM Alexa 594 (Invitrogen) with pH 7.3 and 300 mOsm.

A custom-built two-photon microscope (MOM, Sutter) with a Ti:sapphire laser (Vision II, Coherent), GaAsP photomultiplier tubes, and a 25x water (NA 1.05, Olympus) objective lens was used to acquire a 3D image stack of the Alexa-filled Purkinje cell with a z-step size of 0.25 μm and an xy field of view of 1024 × 1024 pixels. Next, the acquired 3D image stack was deconvoluted using AutoQuantX2 software (Media Cybernetics) using a theoretical point spread function (1–5 iterations) based on specifications from the image acquisition parameters. Later, the dendrite of Purkinje cell was reconstructed with Neurolucida, MBF Bioscience, (http://www.mbfbioscience.com/neurolucida). A different reconstruction of the same Purkinje cell has previously been used in Anwar et al. (2013).

### Ca^2+^ spiking model

The detailed model of spontaneous Ca^2+^ spike generation was derived from the original biophysical model (Schmidt et al., 2003; Anwar et al., 2012) developed in the NEURON simulator (Hines and Carnevale, 1997). The model contained four types of ion channels: P-type Ca^2+^ channel (P_max_ = 2 × 10^−4^ cm/s, GHK equation) (Swensen and Bean, 2005), T-type Ca^2+^ channel (P_max_ = 8 × 10^−6^ cm/s, GHK equation) (Iftinca et al., 2006), BK-type Ca^2+^-activated K^+^ channel (G_max_ = 7 × 10^−2^ S/cm^2^) (Cox et al., 1997) and SK-type Ca^2+^-activated K^+^ channel (G_max_ = 3.1 × 10^−4^ S/cm^2^) (Hirschberg et al., 1998; Solinas et al., 2007), plus a leak channel (G_max_ = 1 × 10^−6^ S/cm^2^ and E_rev_ = −61 mV).

### Ca^2+^ buffering models

Intracellular Ca^2+^ was modeled using the following Ca^2+^ buffering mechanisms.

#### Ca^2+^ pool

The exponential decaying Ca^2+^ pool was modeled as

(1) $$\frac{\text{d}{\left[{\text{Ca}}^{2+}\right]}_{\text{i}}}{\text{dt}}=-\frac{{\text{I}}_{{\text{Ca}}^{2+}}(\text{t})}{2{\text{Fd}}_{\text{eq}}}-\beta ({\left[{\text{Ca}}^{2+}\right]}_{\text{i}}-{\left[{\text{Ca}}^{2+}\right]}_{0})$$

where [Ca^2+^]_i_ is intracellular Ca^2+^ concentration, [Ca^2+^]_0_ is Ca^2+^ concentration at rest and is 45 nM, I_Ca_(t) is total Ca^2+^ current per unit area through VGCC, F is the Faraday's constant, d_eq_ is the equivalent depth of a submembrane shell to define the volume for effective Ca^2+^ concentration, and β is the decay time constant. The values for depth (d) and β, 0.169 μm and 6.86 ms^−1^ respectively, were obtained from a past study (Anwar et al., 2012), where these values were fitted to generate dendritic Ca^2+^ spikes.

Two different definitions for d_eq_ were used. The first definition (SP_old_), uses a mechanism widely used in multi-compartment modeling studies using NEURON (e.g., Miyasho et al., 2001; Poirazi et al., 2003; Hemond et al., 2008; Hay et al., 2011) that takes the volume of the submembrane shell to be directly proportional to its depth d and therefore d_eq_ = d. This results in an incorrect volume of submembrane shell (see details in Results). The second definition (SP_new_) used in this study computed an equivalent depth (d_eq_) for each submembrane shell, which gives the correct volume (see details in Results) when used in the mechanism described by (1):

(2) $${\text{d}}_{\text{eq}}=\text{d}-\frac{{\text{d}}^{2}}{\text{diam}}$$

where diam is the diameter of each compartment.

#### Detailed Ca^2+^ dynamics

The detailed Ca^2+^ dynamics model used in this study was obtained from our previous study (Anwar et al., 2012). It included calbindin (CB) and parvalbumin (PV) as buffers. In addition to Ca^2+^, both PV and 80% of CB were diffusible (Schmidt et al., 2005; Anwar et al., 2012). A single surface-based Ca^2+^ pump was modeled using Michaelis-Menten kinetics (Sala and Hernandez-Cruz, 1990) as follows:

$$\text{pump}+{\text{Ca}}^{2+}\;\overset{{\text{k}}_{\text{b}}}{\underset{{\text{k}}_{\text{f}}}{⇌}}\text{pump}-{\text{Ca}}^{2+}\overset{{\text{k}}_{\text{ext}}}{\to }\text{pump}$$

where pump density was 1 × 10^−15^ mol.cm^−2^, k_f_ was 3 × 10^3^ mM^−1^.ms^−1^, k_b_ was 17.5 ms^−1^ and k_ext_ was 72.55 ms^−1^.

#### Diffusion in NEURON simulator

**Figure 1:**
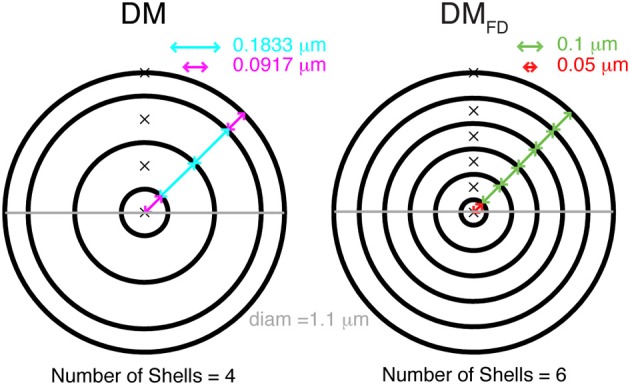
**Cytosolic compartmentalization for diffusion from membrane toward the center of a cylindrical compartment**. Schematic diagram shows two different ways of dividing the compartment volume into concentric shells. The DM mechanism **(left)** has a fixed number of shells and the depths of all shells vary so that the sum equals the compartment diameters, the outer shell also has a smaller depth than subsequent shells. In the DM_FD_ mechanism **(right)** all shells have an identical, fixed depth except for the core shell whose depth is adjusted to get the correct compartment diameter. The number of shells is given by compartment diameter.

In NEURON (Hines and Carnevale, 1997) simulations, diffusion of Ca^2+^, free and bound buffers was allowed only in the radial dimension, i.e., from membrane toward the center of the compartment and vice versa. Two different ways of discretizing space into concentric cylindrical shells were used. The first one, the variable depth scheme, is described as the standard example in the NEURON book (Carnevale and Hines, 2006). Each compartment is subdivided into radial shells (Figure 1) and the number of shells is computed using:

(3) $$\text{Shells}=\left\lfloor \frac{\text{diam}}{4\text{d}}+1.5\right\rfloor$$

where Shells is the number of radial shells, diam is diameter of the compartment and d is depth of the outer radial shell, which was 0.1 μm. The discretization of the compartment volume into radial shells, where the depth of inner radial shells is twice the depth of outer radial shell, resulted in a varying depth of all shells, depending on the diameter of the compartment. The depth (d_1_) of the outer shell and the inner most shell is then:

(4) $${\text{d}}_{1}=\frac{\text{diam}}{4(\text{Shells}-1)}$$

and the other shells have a depth of 2 × d_1_ (see Figure 1).

We also implemented a fixed depth scheme, where all the radial shells except the inner most core shell had a constant depth (Figure 1). The number of shells was computed using:

(5) $${\text{Shells}}_{\text{FD}}=\left\lceil \frac{\text{diam}}{2\text{d}}\right\rceil$$

Here d_1_ as well as the depth of other shells was always 0.1 μm, and the core shell had a variable depth (≤0.1 μm). Note that to model radial diffusion with a variable number of shells, a separate mechanism with a unique configuration of shells for every compartment with a different diameter needs to be created in NEURON.

#### Diffusion in STEPS simulator

To allow 3D diffusion in the stochastic reaction-diffusion simulator STEPS (Hepburn et al., 2012), the dendritic morphology (part of PC 1) was discretized into tetrahedral mesh using CUBIT (http://cubit.sandia.gov).

### Computer simulations

All the simulations were run using a time step of 0.02 ms. Model scripts for all models used in this work are available at http://senselab.med.yale.edu/modeldb/ShowModel.asp?model=155731.

#### Ca^2+^ spike generation in realistic morphologies

Spontaneous Ca^2+^ spikes were generated using realistic morphologies of PCs with ion channels uniformly distributed over the dendrites. The Ca^2+^ spike generation model was simulated with the following conditions: temperature of 34 Celsius, initial voltage of −60 mV, membrane capacitance of 1.12 μF.cm^−2^ and axial resistance of 250 Ohm.cm.

#### Ca^2+^ transients in single compartments

Ca^2+^ transients were simulated using different Ca^2+^ buffering models in single compartments with diameter varying from 0.1 to 6 μm in steps of 0.1 μm. The P-type Ca^2+^ channel with P_max_ of 5.2 × 10^−5^ cm/s was included in the model for Ca^2+^ influx. A “ramp-like” voltage step protocol (same as in Anwar et al., 2012) was used to depolarize the compartment to the voltage at which physiological dendritic Ca^2+^ spikes are generated.

#### Ca^2+^ transients in part of dendritic morphology

Ca^2+^ transients in a part of PC 1 dendritic morphology were simulated using the detailed Ca^2+^ dynamics model with 1D diffusion in NEURON and with 3D diffusion in STEPS. Because of the long runtime for 3D diffusion simulations it was not possible to simulate a complete PC in STEPS.

A uniform current (in mA/cm^2^) recorded during a Ca^2+^ spike was applied to each compartment in the NEURON simulations to evoke a constant shape of the spike. Two types of compartmentalization approaches were used in these simulations. Firstly, using a single compartment per dendritic section (Total sections = 45). Secondly, each of the dendritic section was split into multiple (1–22) sections (Total sections = 300), where each section consisted of adjacent traced points on the dendrite.

Ca^2+^ influx in STEPS was implemented using first-order surface based Ca^2+^ influx reaction (X 𠆒 X + Ca^2+^), where “X” channels (100,000) were distributed uniformly over the surface triangles (~92,000) of the mesh. At each time point, the influx rate per channel was updated based on the Ca^2+^ influx profile (obtained using the total current applied in the NEURON simulation). Due to high rate and uniformity of influx, effects of stochasticity were negligible. The results of STEPS simulations in this study are reported as the mean computed over 10 trials.

## Results

**Figure 2:**
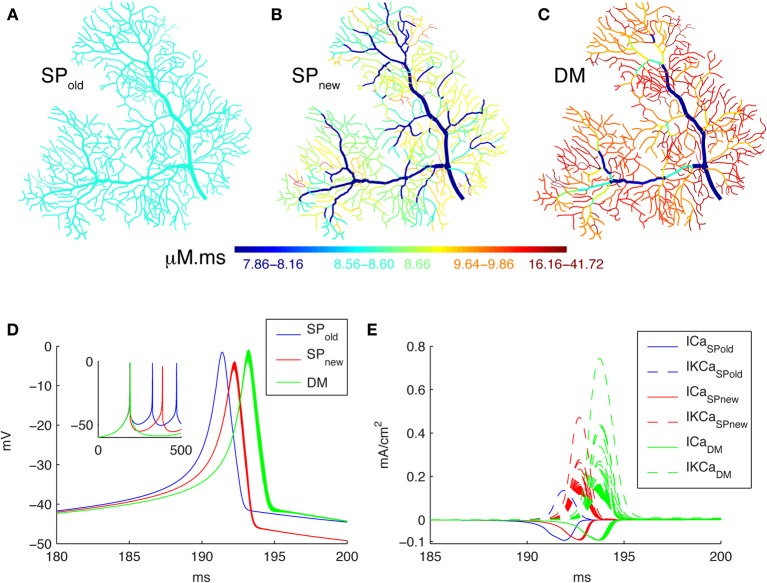
**Spatial Ca^2+^ gradients strongly depend on type of model implementation**. Panels **(A–C)** show maps of the integrated calcium levels in the dendrite during a spontaneous burst of Ca^2+^ spikes (panel **D**). The dendritic branches are color coded to show the integrated calcium levels using a 20 ms window around the peak Ca^2+^ concentration of the first dendritic Ca^2+^ spike. The color scales used in these maps are nonlinear (using histogram equalization) to enhance the contrast. **(A)** Single Ca^2+^ pool model using SP_old_ mechanism results in homogenous Ca^2+^ levels. **(B)** Single Ca^2+^ pool model using SP_new_ mechanism results in variable Ca^2+^ levels. **(C)** Detailed Ca^2+^ dynamics model with buffering and 1D diffusion results in variable Ca^2+^ levels with larger Ca^2+^ gradients. **(D)** Voltage traces show the first spike of the Ca^2+^ burst for each model in all dendritic compartments for the 3 different models (see color code in Figure). The inset shows complete traces. **(E)** The underlying Ca^2+^ and K_Ca_ currents (recorded from all dendritic compartments) for the Ca^2+^ spike of the three different models (see color code in Figure).

In this study we explore the effect of dendrite diameter on Ca^2+^ dynamics in models of different complexity. Figure 2 shows that simulation results are strongly influenced by how one implements the model by comparing the integrated Ca^2+^ concentrations (for all time points in a time window, the sum of Ca^2+^ concentrations multiplied by the time step) in three different models of a spontaneous burst of Ca^2+^ spikes (Figure 2D; see the corresponding currents in Figure 2E) computed using the NEURON simulator. Figure 2A shows the result when using a simple pool model to compute Ca^2+^ concentrations based on an approach used in most NEURON simulations (SP_old_, e.g., Miyasho et al., 2001; Poirazi et al., 2003; Hemond et al., 2008; Hay et al., 2011). Using this approach no gradients of Ca^2+^ concentration are predicted within the dendrite; this result is unlikely to be physiological considering the large variation in SVR across the dendrite. The model in Figure 2B (SP_new_) also uses a simple pool but implemented differently; it results in strong Ca^2+^ gradients with higher concentrations in thin dendritic branches as expected from the SVR. Finally we simulated a detailed Ca^2+^ dynamics models with buffers and radial 1D diffusion (Figure 2C, DM). This shows similar gradients as SP_new_, but with higher Ca^2+^ peak values as expected from previous work comparing DM to simple pool models (Anwar et al., 2012).

Next we will describe in detail the differences between SP_new_ and SP_old_ and then analyze the diameter dependence in SP_new_ and DM.

### Inaccuracy of Ca^2+^ volumes in simple pool models and their effects on Ca^2+^ levels

**Figure 3:**
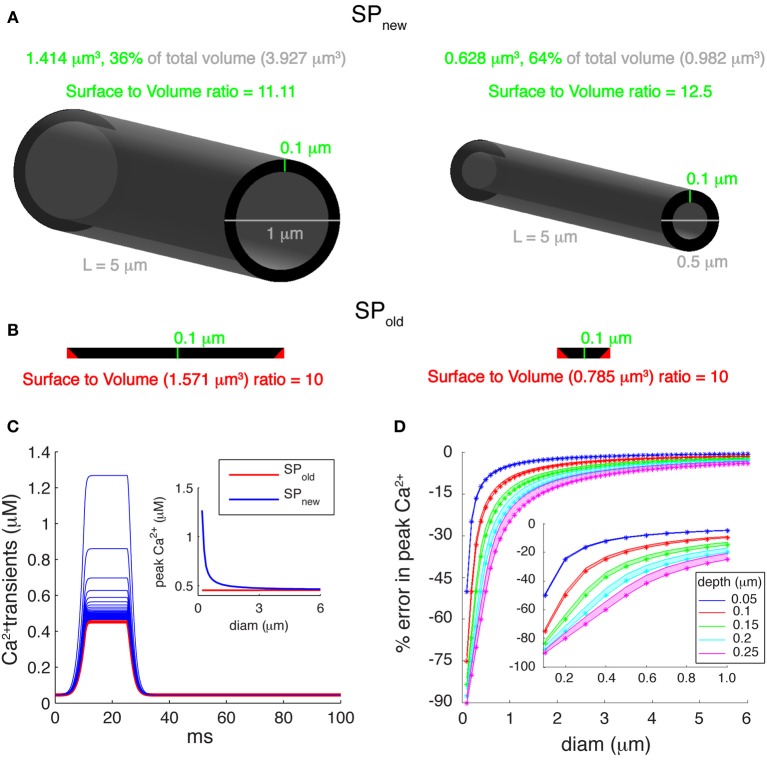
**Errors introduced by incorrect submembrane volumes of single pool models. (A)** Comparison between cylindrical dendritic compartments with diameters of 1 μm (left) and 0.5 μm (right) with submembrane shells with a depth of 0.1 μm. A correct implementation of the volume of the submembrane shell representing the single Ca^2+^ pool (SP_new_ mechanism) results in a SVR that depends on the compartment diameter. **(B)** For the same compartments using the SP_old_ mechanism results in volumes that are too large and have a constant SVR. The cross-sectional area of each compartment (black disks shown in **A**) is unfolded and drawn to show that the actual volume of the submembrane shell (SP_new_) is smaller than the volume used in the SP_old_ mechanism. The red triangles represent extra cross-sectional area included in the volume of SP_old_. **(C)** Ca^2+^ transients generated using a “ramp-like” voltage command in single compartments with diameters ranging from 0.2 to 6 μm in steps of 0.1 μm. P-type Ca^2+^ channel with P_max_ of 5.2 × 10^−5^ cm/s was used for Ca^2+^ influx. Inset: comparison of peak amplitudes of Ca^2+^ transients using SP_old_ and SP_new_ show that the first mechanism causes exactly the same transient in all compartments, whereas, SP_new_ causes transients with varying peak Ca^2+^ amplitudes. **(D)** Error in peak Ca^2+^ levels caused by using the SP_old_ mechanism [error = (max([Ca^2+^]_SP_old_) − max([Ca^2+^]_SP_new_)/max([Ca^2+^]_SP_new_))]. Pool models used β-values of 0.02, 6.86, and 10 ms^−1^; and depth (d) values of 0.05, 0.1, 0.15, 0.2, and 0.25 μm. The lower edge of shaded areas of each color shows error in peak calcium for β-value of 10 ms^−1^, whereas, the upper edge of shaded areas of each color show error for β-value of 0.02 ms^−1^. The colored asterisks show corresponding error for β-value (used to model PC dendrites) of 6.86 ms^−1^. Inset highlights large errors for branches with small diameters (diam ≤ 1 μm).

Many multi-compartment dendritic models use a single pool model of Ca^2+^ buffering, which simulates only the submembrane Ca^2+^ concentration to control K_Ca_ channels. These phenomenological models convert Ca^2+^ current passing through VGCC to Ca^2+^ concentration using a submembrane shell of fixed depth, d [Equation (1); Figure 3A]. The volume of a submembrane shell (as for SP_new_) is defined as:

(6) $${\text{Vol}}_{\text{s}\_\text{new}}={\text{Vol}}_{\text{f}}-{\text{Vol}}_{\text{c}}=\pi \text{d}(\text{diam-d})\text{L}$$

where Vol_f_ denotes the volume of a full compartment, Vol_c_ denotes the volume of the core, d is the depth of submembrane shell, diam is the diameter of compartment and L is its length. Using such a representation of submembrane shell, SVR_new_ equals

(7) $${\text{SVR}}_{\text{new}}=\frac{\text{SA}}{{\text{Vol}}_{\text{s}\_\text{new}}}=\frac{\text{diam}}{\text{d}(\text{diam}-\text{d})}$$

where SA is the surface area of the compartment. Note that SVR_new_ is less dependent on diameter than the SVR for the complete volume (1/diam), but, as shown in Figure 3A, SVR_new_ still increases for smaller diameters.

However, we noticed that most of the Ca^2+^ shell models implemented in NEURON use an incorrect volume for the submembrane shell (Figure 3B). In those models (SP_old_), the volume of a submembrane shell is defined as:

(8) $${\text{Vol}}_{\text{s}\_\text{old}}=\text{SA}\times \text{d}=\pi \text{diamLd}$$

Using such a representation of submembrane shell, gives SVR_old_

(9) $${\text{SVR}}_{\text{old}}=\frac{\text{SA}}{{\text{Vol}}_{\text{s}\_\text{old}}}=\frac{1}{\text{d}}$$

Because the depth (d) of submembrane shells is usually taken constant for variable diameter compartments, SVR_old_ is constant and independent of the dendrite diameter. As a result, we observe changes in peak amplitudes of the simulated Ca^2+^ transients using SP_new_ in compartments with different diameters (Figure 3C) while the same compartments with SP_old_ always show exactly the same Ca^2+^ transient.

Since pool based models are phenomenological models, the values of depth (d) and decay time constants (β) can be tuned to approximate the desired behavior of intracellular [Ca^2+^]. In Figure 3D we show how the error of using SP_old_ (compared to SP_new_) depends on the values of d and β used. The errors in peak Ca^2+^ were computed using β-values of 0.02 ms^−1^ (Traub and Llinas, 1977), 6.86 ms^−1^ (Anwar et al., 2012), and 10 ms^−1^ (De Schutter and Bower, 1994) and using submembrane shells with depths ranging from 0.05 to 0.25 μm. The error increases with the size of depth used, as expected from SVR_new_. More importantly, these errors become significantly larger for smaller diameters (diam <1 μm) and may reach up to 80% for 0.1 μm diameter compartments (inset of Figure 3D). Typically, distal dendrites have large numbers of dendritic branches with diameters less than 1 μm.

In the rest of our study we will only focus on SP_new_ and DM to investigate how well they can capture Ca^2+^ gradients in dendrites.

### Detailed Ca^2+^ dynamics model cause large spatial variability of Ca^2+^ levels in realistic dendritic morphologies

**Figure 4:**
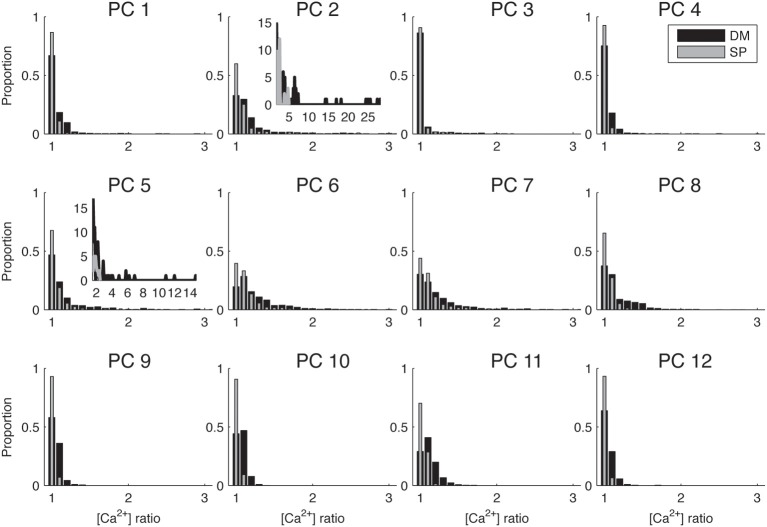
**Biophysically detailed Ca^2+^ dynamics model causes larger differences in calcium levels in adjacent dendritic branches than single pool models**. Histograms of ratios between integrated calcium from adjacent dendritic branches for 12 different PCs using SP_new_ and DM. To make the differences between cells more visible only the range of ratios 1–3 is shown, for the two cells that have significantly larger ratios the full distribution is shown in the inset. PC1 is shown in Figure 2. Integrated Ca^2+^ was computed for 20 ms around the first peak of Ca^2+^ transients for all PCs.

Figure 2 demonstrates in one dendritic morphology that SVR differences cause sharper Ca^2+^ gradients when it was modeled using DM compared to SP_new_. We next investigated whether this is a systematic observation by simulating the dendritic Ca^2+^ spike model in 11 additional dendritic reconstructions of PCs using both methods and comparing the results (Figure 4). For each PC, the ion channels were distributed uniformly on its dendrite and each unbranched segment had a constant diameter.

Because this study mostly focuses on local differences in Ca^2+^ concentration, we summarized the data on spatial gradients by computing the ratio of integrated [Ca^2+^] in adjacent dendritic segments and plotting the distributions of these ratios in Figure 4. We observe a wide range of distributions of spatial fluctuations of Ca^2+^ levels in different neuron reconstructions, with the histograms of some neurons (e.g., PC 2 and 5) showing very large tails and other ones only small fluctuations (ratio < 2). This observation may be related to differences in the quality of the reconstructions (see Discussion). But in all cases, the DM model always results in a wider range of Ca^2+^ fluctuations as compared to SP_new_.

### Variability of diameters in morphological reconstructions of neurons

**Figure 5:**
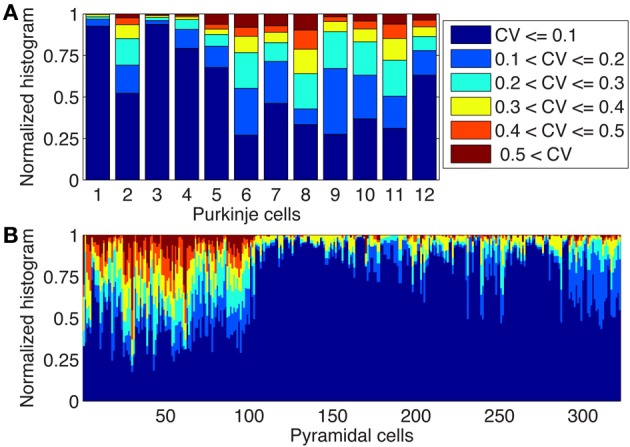
**Large changes in diameters of unbranched dendritic segments exist in Purkinje and Pyramidal neurons**. Stacked histograms show the distribution of CV values for the diameters changes over unbranched dendritic segments in Purkinje cells (**A**, *N* = 12) and in neocortical and hippocampal pyramidal neurons (**B**, *N* = 322). Notice the presence of large variability of diameters (CVs > 0.2 or more) in many neurons and the large neuron to neuron differences which are mostly caused by lab to lab differences in reconstruction quality (see text).

In Figure 4 we used a common approach in compartmental modeling: we ignored small variations in diameter by taking only a single compartment for each unbranched segment. However, due to the large jumps in Ca^2+^ concentrations between neighboring compartments observed in some parts of the model (Figures 2, 4) we wondered about the realism of this assumption of uniform diameter. We investigated this issue both in the 12 PCs modeled previously as well as in 284 neocortical and 38 hippocampal pyramidal neuron reconstructions, because larger changes in diameter may be present in morphological classes where the level of branching is not as extensive as in PCs. For both neuron types we computed the coefficient of variation (CV) of diameters for every dendritic segment (between two branch points) based on all the measurements available in the morphological reconstruction (Figure 5). We observed a large variability in CV of reconstructed morphologies of neurons obtained from different laboratories for both cell classes (see Discussion), but overall the variability of diameter was much larger in pyramidal neurons where in many cells more than a quarter of the unbranched segments had CVs of 0.4 or more. In PCs more than half of the reconstructions had CVs of 0.2 or more in at least a quarter of their unbranched segments.

Our analysis suggests that for pyramidal neurons even more care should be taken when converting dendritic segments into cylindrical compartments. A good representation of dendritic segments with rapidly varying diameters is essential to model intracellular Ca^2+^ concentrations correctly (see also **Figure 8**).

### Effect of volume discretization on detailed Ca^2+^ dynamics models

Though the implementation of 1D diffusion in concentric cylindrical shells may seem straightforward, the NMODL language used in NEURON actually makes it difficult to do this in a flexible way and we discovered that many existing models do not implement it correctly. The standard example in the NEURON book (Carnevale and Hines, 2006) is a variable depth scheme where the volume is divided over a fixed number of concentric shells (4 in the standard example) with variable depth Equations (3) and (4) and Figure 1: DM, note that the submembrane and core shells have a smaller depth than the others). Many models using NEURON implement exactly this mechanism: 4 shells and all with variable depth. Because the volume of the submembrane shell is used to convert inward Ca^2+^ currents into a Ca^2+^ concentration that directly activates K_Ca_ channels, varying its depth will affect the computed value of this Ca^2+^ concentration. From a biophysical perspective there is no reason why the depth of a submembrane shell that is assumed to simulate the effective volume affecting the Ca^2+^ sensors of K_Ca_ channels (Fakler and Adelman, 2008; De Schutter, 2010) should vary greatly with dendrite diameter. We will therefore consider two issues: the number of shells to be modeled and a submembrane shell with variable (DM) or fixed depth (DM_FD_).

**Figure 6:**
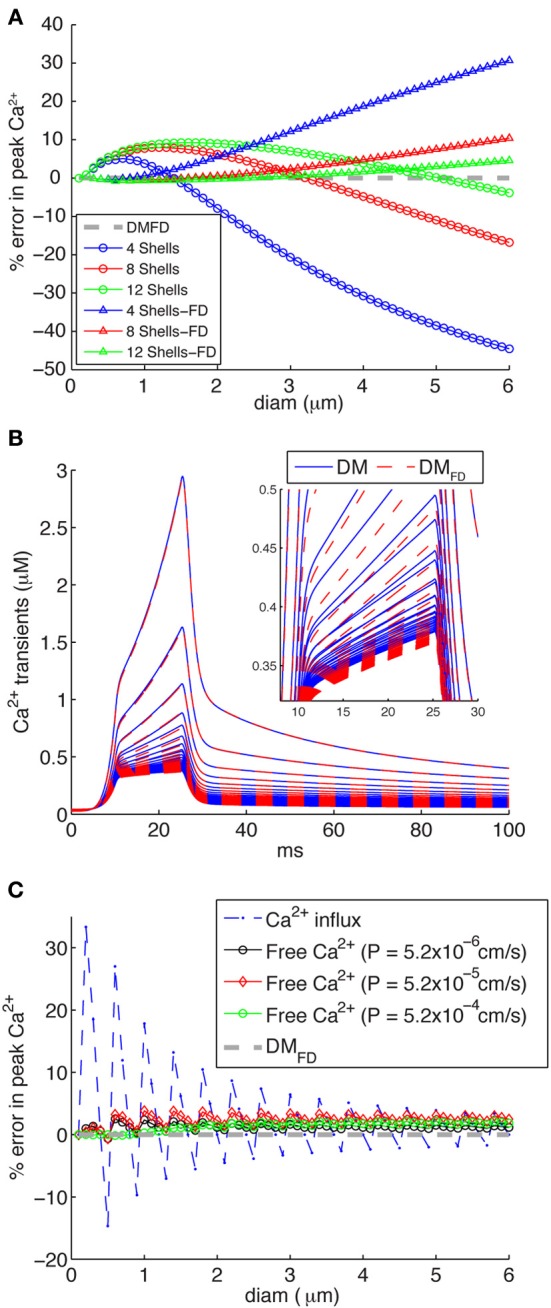
**Inaccuracies of different calcium 1D diffusion models result in erroneous calcium levels. (A)** Errors introduced by making the number of concentric shells independent of compartment diameter, for 4, 8, or 12 shells respectively. Two mechanisms are implemented: the standard NEURON scheme with variable depths for all shells (circles) and an FD scheme where the submembrane shell has a constant depth d_1_ = 0.1 μm and the rest of the shells has variable depth (triangles). The DM_FD_ mechanism is used as reference. Note that for both mechanisms the errors become large for diameters beyond 2 μm if only four shells are used (as is the case in some NEURON models). **(B)** Ca^2+^ transients generated using a “ramp-like” voltage command in single compartments (see Figure 3C for details) comparing the responses of the DM and DM_FD_ models. Both models show very similar behavior with only small numerical differences. **(C)** Errors due to discretization of radial shells in DM, which may result in variable d_1_ resulting in rapid changes of submembrane shell volume for increasing compartment diameter. The broken line with asterisks shows errors related to conversion of Ca^2+^ influx to Ca^2+^ concentration with variable depth d_1_ of the submembrane shell (it varies between 0.075 and 0.125 μm due to discretization) as compared to fixed d_1_ of 0.1 μm (DM_FD_). The solid lines with diamonds shows the actual error in free Ca^2+^ in the submembrane shell for DM models for different sizes of Ca^2+^ influx as indicated. Note that these errors are much smaller than predicted by the Ca^2+^ influx conversion.

If one wants to vary the number of shells modeled depending on compartment diameter, which is the correct solution, a separate NEURON mechanism has to be created for each specific number of shells that is required. Some authors have therefore decided to use a fixed number of shells with variable depth of each shell (Migliore et al., 1995; Lazarewicz et al., 2002; Gold et al., 2007; Lavzin et al., 2012) (Figure 1: DM), but this can lead to significant errors in simulated submembrane Ca^2+^ concentration in large diameter dendrites if the number of shells is taken to be small (Figure 6A: circles). These errors show both a positive and negative component depending on compartment diameter, suggesting that two types of error contribute. Indeed, when we repeated these simulations with a fixed submembrane shell depth d_1_ of 0.1 μm and the rest of the volume divided over the remaining shells with equal, variable depths (FD: Figure 6B: triangles) only a positive error, increasing with diameter, remains. Because this error is quite small for a large number of shells, a model with the same large number of shells in every compartment will give accurate results in NEURON, but this may cause unacceptably slow runtimes (Anwar et al., 2012) so it is better to vary the number of shells [DM_FD_ mechanism, Equation (5)].

The next question is then how to compute shell depth as the depth of at least one shell has to vary to fit the total exactly to a variable compartment diameter. As already mentioned, in the standard NEURON implementation (Carnevale and Hines, 2006) the depths of all shells vary with compartment diameter [variable depth scheme; Equations (3) and (4) and Figure 1: DM], including that of the submembrane shell. In effect, the depth of the submembrane shell (d_1_) may vary between d − 0.25 d and d + 0.25 d. In Figure 6C, the broken line shows the theoretical error of Ca^2+^ influx conversion to Ca^2+^ concentration using the variable depth scheme (range of d_1_ due to discretization: 0.075–0.125 μm). The larger predicted errors in these cases are associated with small diameters, where small changes of diam will result in bigger changes in d_1_ Equation (4) and submembrane shell volume.

These errors are large and should not be ignored. But what is the effect of these geometrical errors on actual computed Ca^2+^ concentrations? To quantify this we simulated Ca^2+^ transients using a mechanism with a variable number of shells, all with the same depth of 0.1 μm except for the core shell which has a variable diameter [DM_FD_, Equation (5) and Figure 1], which is assumed to give the most accurate solution. We found that DM and DM_FD_ show very similar peak amplitudes and decay time constants for different diameter compartments (Figure 6B), resulting in much smaller errors in peak amplitudes of Ca^2+^ using DM compared to DM_FD_ than theoretically predicted (Figure 6C). The error depends on the size of Ca^2+^ influx in a nontrivial way, but for all levels of Ca^2+^ influx it was small with the largest error only about 4%. This significant difference with the theoretical prediction is due to strong buffering (especially in PCs; (Hartmann and Konnerth, 2005) and diffusion of Ca^2+^, which removes most of Ca^2+^ entering into the submembrane shell.

**Figure 7:**
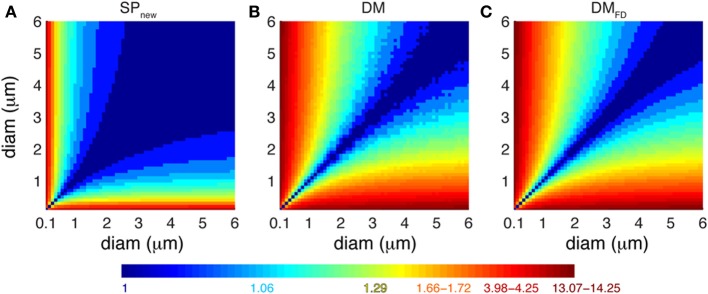
**Different Ca^2+^ buffering model respond variably to changes in dendrite diameters**. Predicted ratio of integrated Ca^2+^ concentration (100 ms window) for different combinations of diameters of pairs of dendritic compartments using **(A)** SP_new_, **(B)** DM, and **(C)** DM_FD_. The maps are derived from the data shown in Figure 3C **(A)** and Figure 6B **(B,C)**. The color scales used in these maps are nonlinear (using histogram equalization) to enhance the contrast.

How do the different Ca^2+^ buffering models respond to local fluctuations of dendrite diameter in terms of Ca^2+^ levels? To estimate the effect of dendritic diameter changes on Ca^2+^ dynamics using each model, we computed the ratio of integrated Ca^2+^ transients measured in each of the pair of simulated compartments using SP_new_, DM and DM_FD_ for many possible pairs of dendritic diameters (range: 0.1–6.0 μm with increments of 0.1 μm) (Figure 7). As explained previously, the SP_old_ model does not show any sensitivity to changes in diameters. For SP_new_, large ratios (>2) are limited to combinations where a compartment with an extremely small diameter (≤0.3 μm) is connected to one with large diameters. For DM and DM_FD_, this region expands to all compartments with diameter less than or equal to 1 μm that are connected to ones with larger diameters. Therefore, the detailed Ca^2+^ dynamics models are more sensitive to changes in dendritic diameter compared to pool based models, which explains the differences observed in Figure 3. But, although the sensitivity maps of DM are noisier than those of DM_FD_, due to the use of variable depth submembrane shells in DM, overall these maps are quite similar to each other.

We conclude from Figures 6, 7 that correct simulation of radial 1D Ca^2+^ diffusion requires a variable number of concentric shells that scales with compartment diameter, but that the Ca^2+^ dynamics are less sensitive to the actual scheme used to compute the depth of these shells.

### Spatial differences in Ca^2+^ levels persist with 3D diffusion

The predictions of Figure 7 are based on no (SP_new_) or only radial 1D diffusion (DM and DM_FD_). In this case, would the predicted large Ca^2+^ transients disappear in the presence of 3D diffusion? Also, what happens if dendritic diameter varies more smoothly than possible in a model using electrical compartmentalization? To address both issues, we used the STEPS simulator (Hepburn et al., 2012), which uses tetrahedral meshes to accurately represent detailed morphologies and 3D diffusion of molecules to simulate Ca^2+^ dynamics.

**Figure 8:**
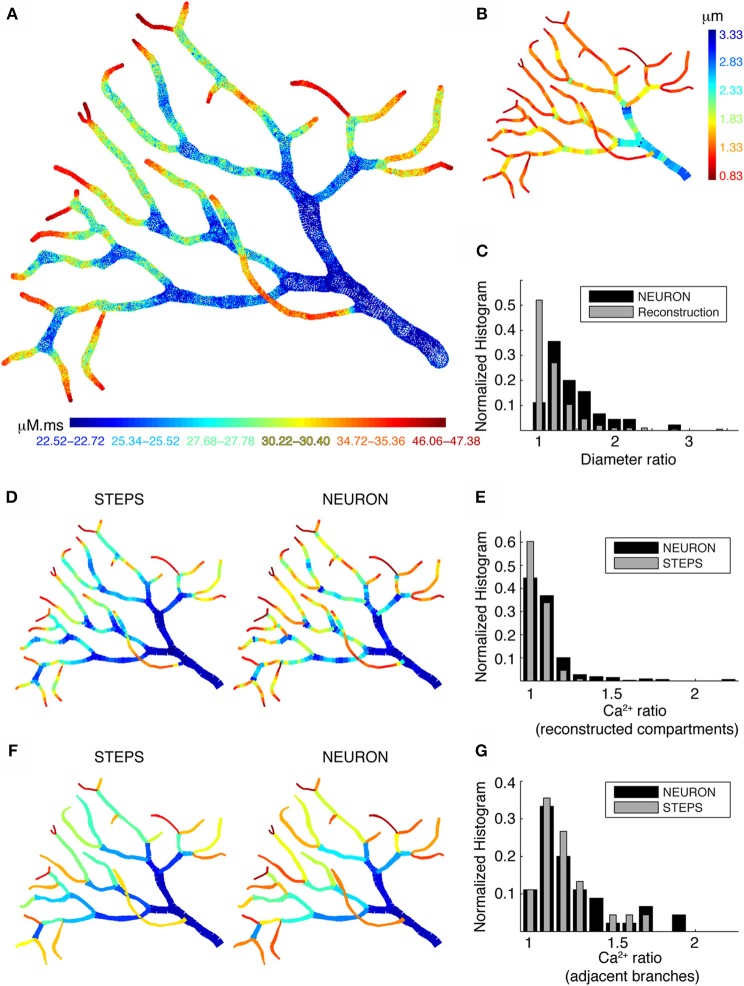
**Large differences in calcium levels in adjacent dendritic branches persist in presence of 3D diffusion. (A)** STEPS model using 3D buffered diffusion to compute the Ca^2+^ concentration resulting from the burst of Ca^2+^ spikes. Spatial map of integrated calcium (140 ms window) in a piece of carefully reconstructed PC dendritic arbor (part of PC 1). Every colored dot drawn at the center coordinates of each tetrahedron belonging to the mesh in which 3D diffusion was simulated shows the integrated Ca^2+^ in that particular tetrahedron. Only tetrahedrons representing the submembrane region are plotted. The color scales used in these maps are nonlinear (using histogram equalization) to enhance the contrast. **(B)** Spatial map of dendritic diameters in the dendrite shown in **(A,D,F)**. **(C)** Normalized histograms compare the ratios of adjacent diameters in the original morphological reconstruction with similar ratios of diameters of adjacent compartments in the NEURON model (1 segment per unbranched section). **(D,E)** NEURON simulation with many compartments for each unbranched segment, carefully reflecting the variability of dendrite diameter. Data for the STEPS simulation are averaged over all tetrahedrons representing the corresponding NEURON compartment. **(F,G)** NEURON simulation with a single compartment for each unbranched segment, data for the STEPS simulation averaged for corresponding NEURON compartments. **(D,F)** Spatial maps of integrated submembrane Ca^2+^ concentration using the detailed calcium dynamics model with 3D diffusion (STEPS) and 1D radial diffusion (NEURON) are shown for the different compartmentalization schemes. **(D)** and **(F)** Use same color as in **A**. **(E,G)** Normalized histograms show the ratios of integrated Ca^2+^ concentration between every adjacent compartment using simulations with 3D diffusion (STEPS) and 1D diffusion (NEURON) for the results shown in **(D,F)** respectively.

When we simulated Ca^2+^ transients with the detailed Ca^2+^ dynamics model in part of a PC dendritic arbor using STEPS, we still observed large fluctuations in Ca^2+^ levels along the different branches. Figure 8A shows the integrated Ca^2+^ levels for each tetrahedron located within 0.1 μm from the membrane. The large fluctuations of Ca^2+^ levels appear to be related to dendrite diameter. High Ca^2+^ levels are observed in dendritic regions with small diameter (Figure 8B) and at the tips of terminating branches. Higher levels at the tips are due to the higher SVR as a result of their small diameters and the reduced effective diffusion because of the closed end condition (the latter is not predicted by radial diffusion models). Overall we conclude that neither 3D diffusion nor smooth changes in dendrite diameter reduce the pronounced Ca^2+^ gradients caused by variable dendrite diameter, raising the question what level of detail is necessary to model this effect correctly?

To address this question, we compare NEURON and STEPS simulations using two different compartmentalization schemes in NEURON. First, we simulated Ca^2+^ transients in NEURON using multiple compartments per unbranched segment to capture all changes in dendritic diameters (Figure 8D; right panel). For comparison Figure 8D (left panel) shows the STEPS simulation with mean integrated Ca^2+^ concentration computed for all tetrahedrons corresponding to every NEURON compartment. Next, we made a similar comparison with NEURON simulations where every unbranched dendritic segment is considered as a single compartment (Figure 8F), which is the approach used in many compartmental models. Comparing these spatial maps (Figures 8D,F), we observe only small differences between simulations with 1D diffusion (NEURON) or with 3D diffusion (STEPS). However, the actual Ca^2+^ levels are different in the respective simulations. To quantify the difference in Ca^2+^ levels between the two approaches and how they relate to fluctuations in dendritic diameters we computed the ratios of Ca^2+^ levels and diameters for all adjacent segments. Figure 8E shows Ca^2+^ ratios in adjacent compartments for small compartment sizes (data shown in Figure 8D) and Figure 8G shows Ca^2+^ ratios in adjacent compartments with one compartment per unbranched dendritic segment (data shown in Figure 8F). The comparison of Figure 8E with Figure 8G clearly shows that the use of large compartments will result in larger jumps in Ca^2+^ levels between adjacent compartments. Using many small compartments to capture the continuous change of dendritic diameters results in much smoother and smaller changes in Ca^2+^ levels. The overall behavior of these changes in Ca^2+^ levels (Figures 8E,G) is similar, respectively, to the ratios of diameters in the original morphological reconstruction and to the ratios for adjacent compartments diameters for one compartment per unbranched segment (Figure 8C). This confirms that the simulated Ca^2+^ gradients are largely caused by the SVR effect. Finally, notice that the effect of 3D diffusion is more prominent when using small compartments (Figure 8D, bigger difference between NEURON and STEPS simulation).

## Discussion

For a long time (until early 1960s), dendrites were thought to be passive structures, whose main function was to transfer and sum information from presynaptic to postsynaptic neurons (for review see Johnston et al., 1996). During the past couple of decades, it has been shown that dendrites contain a variety of voltage-gated channels (Llinas et al., 1992; Markram and Sakmann, 1994; Stuart and Sakmann, 1994; Magee and Johnston, 1995; Magee and Carruth, 1999; Lorincz and Nusser, 2010), voltage-dependent NMDA channels (Losonczy et al., 2008; Polsky et al., 2009; Major et al., 2013) and K_Ca_ channels (Golding et al., 1999; Womack and Khodakhah, 2002, 2003), which make these structures active. In addition to their role in neuronal excitability and dendritic integration, dendrites with thousands of synapses also serve as a venue of memory storage through induction of synaptic plasticity. Intracellular Ca^2+^ is involved in many processing capabilities of dendrites. Ca^2+^ entering through VGCC and NMDA channels gives rise to cytosolic Ca^2+^, which in turn activates various K^+^ channels and several molecular signaling pathways underlying synaptic plasticity. Therefore, it is important to correctly understand the dynamics of intracellular Ca^2+^ in dendrites with complex morphological structures.

### Previous modeling of detailed calcium dynamics

The complexity of dendritic geometry and structure has been studied extensively to investigate its effects on propagation of action potentials, its role in synaptic efficacy and its effects on limiting interaction across different active dendritic regions (Mainen and Sejnowski, 1996; Vetter et al., 2001). Although variable levels of Ca^2+^ in different dendritic regions have been reported previously (Tank et al., 1988; Lev-Ram et al., 1992; Schiller et al., 1995), only a few studies specifically investigated the effect of dendritic diameters on Ca^2+^ dynamics (Holthoff et al., 2002; Rozsa et al., 2004). Therefore, those effects are often omitted while constructing biophysical models of dendrites. Due to limited quantitative information about the mechanisms controlling Ca^2+^ levels in many neurons, phenomenological models of Ca^2+^ buffering, such as the single exponential decaying pool, are commonly used for biophysical neuronal modeling. Such models when used correctly capture only some aspects of the highly complex behavior of intracellular Ca^2+^ buffering dynamics. In our previous work (Anwar et al., 2012), we showed that pool based models of Ca^2+^ buffering fail to correctly predict peak Ca^2+^ concentrations and decay time constants important for the interaction between VGCC and K_Ca_ channels. In this study, we investigated the effect of dendritic diameters on Ca^2+^ dynamics using a modeling approach. Our results (Figures 2, 4) show that pool based models have limited ability to capture the spatial variability of Ca^2+^ dynamics in morphologically complex dendrites as compared to a detailed Ca^2+^ dynamics model with radial 1D diffusion. The detailed Ca^2+^ dynamics model shows different peak amplitudes of Ca^2+^ levels as well as different (and multiple) decay time constants (Figure 6B). In contrast, pool based models with correct submembrane volume only show different peak amplitudes of Ca^2+^ levels (Figure 3C).

In general, many studies, including this one, ignore additional properties of real neurons that will affect Ca^2+^ dynamics. The most important of these simplifications are the assumption of constant density of Ca^2+^ channels, which is known to be not true for many neurons (for review see Johnston et al., 1996; Migliore and Shepherd, 2002), and the omission of the effect of organelles in the cytoplasm that block diffusion and have additional membrane Ca^2+^ pumps (mainly endoplasmic reticulum and mitochondria). Another important determinant of Ca^2+^ dynamics is inhomogeneous distribution of Ca^2+^ buffers in dendrites of a given neuron, as well as their properties, causing competitive binding of Ca^2+^ to available Ca^2+^ buffers and Ca^2+^ pumps (Markram et al., 1998). While the density of channels can easily be changed in compartmental models, accurate representation of intracellular organelles is possible in mesh based models only. We do not expect that inclusion of these properties would significantly change our conclusions.

### Importance of accurate morphological reconstruction

Dendrites have variable diameters. Typically, the diameter of dendrites taper with increasing distance from the soma. It is generally assumed that the change in diameter of an unbranched dendrite is relatively small as compared to the change in diameter at branching, which allows representation of an unbranched dendrite segment as a single uniform diameter compartment. However, as shown in our morphological analysis in Figure 5, many reconstructions of both PCs and pyramidal neurons show great diameter variability within their unbranched segments, with CV values sometimes reaching above 0.5. This implies that a correct Ca^2+^ dynamics model should represent this diameter variability by having several compartments for each unbranched segment (Figures 8D–E), but also that the quality of the morphological reconstruction is of utmost importance. We observed great differences of the diameter variability between different neural reconstructions which often could be related to the laboratory where the reconstructions have been done, as was reported previously for pyramidal neurons (Scorcioni et al., 2004; Szilagyi and De Schutter, 2004; Holmes et al., 2006). Because it seems more likely that human error causes an undersampling of diameter changes than an exaggeration, we assume that the reconstruction with high diameter CV tend to be more reliable. Finally, one should be aware that software like CVapp (Cannon et al., 1998), which converts morphology files into formats suitable for NEURON simulation, uses a specific discretization scheme that changes diameters at branch points (Figure S1).

Although morphological reconstructions obtained using electron microscopy (EM) capture dendrites much more precisely, because of rapid fluctuations in dendritic surface those reconstructions are not suitable for compartment based models. The proper use of EM reconstructions in modeling Ca^2+^ dynamics will require more advanced simulators with support for surface or tetrahedral meshes (e.g., M-Cell, STEPS). Also, this will require more detailed description of Ca^2+^ related mechanisms (e.g., spatial distribution of VGCa channels, K_Ca_ channels, buffers, pumps and internal calcium stores).

### Simulator implementation issues

Almost all biophysically detailed models have been constructed using either the GENESIS or NEURON simulators. These software packages are based on compartmentalization of dendritic structures into multiple iso-potential cylinders, where voltage, currents and concentrations are computed for each of those compartments independently. Since these compartments are based on electrical properties of dendrites, biochemical representation of intracellular mechanisms in these compartments is always an approximation of the related biophysical process. Such a simplified molecular representation may result in unrealistic behavior of the model, depending on the rationale behind the assumption and its accuracy. One such example is the commonly used single pool model to simulate intracellular Ca^2+^ in the NEURON simulator Equations (8) and (9). The conversion of influx to intracellular Ca^2+^ concentration in these models is incorrect, which is due to the use of an inaccurate volume of the submembrane shell (Figure 3B). Although this inaccuracy does not influence the results of single compartment models dramatically because it can be easily tuned by adapting the shell depth, it becomes critically important in multi-compartment models. This incorrect single pool model will always underestimate the influx (Figure 3C), which may require unrealistic distribution of dendritic VGCC and K_Ca_ channels during model construction and cause a mismatch in input resistance between model and actual cell, and it will not predict any spatial gradients of Ca^2+^ concentration due to fluctuations of dendrite diameter (Figure 2A).

More detailed Ca^2+^ dynamics models using radial 1D diffusion are thought to be more accurate, but again the compartmentalization of the dendrite may result in either an inaccurate or incomplete representation of model. It is a major challenge to model diffusion in the NEURON simulator correctly. NEURON allows radial (toward the center of each dendritic compartment) and longitudinal (from one compartment to neighboring compartment) diffusion. Radial diffusion requires virtual submembrane shells (Figure 1), where shells typically have a variable depth, depending on the diameter of each compartment. Furthermore, longitudinal diffusion is only allowed if the adjacent compartments have the same number of shells which will introduce a larger error (Figure 6A) unless a very large number of shells is used everywhere. Conversely, though theoretically the variable submembrane depths of the standard NEURON scheme (DM) should result in large errors, this effect was strongly filtered by the diffusion and buffering mechanisms, resulting in only small differences (Figure 6C) with a method (DM_FD_) that ensures a fixed depth of the submembrane shell. It should be noted, however, that these differences may be larger in models of other neurons because the buffering capacity of PCs is exceptionally high (Hartmann and Konnerth, 2005). Control simulations showed that although the extent of the changes in Ca^2+^ levels varied in models with lower buffers concentrations, the dependence of Ca^2+^ levels on changes in diameters persisted (results not shown).

Neither of the issues just mentioned are relevant for the GENESIS simulator. Both submembrane pools (as the concpool object, De Schutter and Bower, 1994) and radial diffusion (as the difshell object, De Schutter and Smolen, 1999) are implemented correctly and are easy to set up. Conversely, it is time consuming to create multiple calcium dynamics mechanisms with radial diffusion in NEURON because a separate mechanism has to be written for each set of diameters (see Materials and Methods) and this requires a lot of extra care. And then, for every different morphology, one will have to repeat the process. We expect that multilevel declarative model description languages (Raikov and De Schutter, 2012) may allow transparent and correct compartment based assignment of molecular mechanisms in NEURON in the future.

### Recommendations for correct modeling of dendritic Ca^2+^ dynamics

Even in PCs, where the estimated Ca^2+^ diffusion range is only about 5 μm (Santamaria et al., 2006), we observe effects of 3D diffusion on Ca^2+^ transients compared to when only radial 1D diffusion is used, especially when Ca^2+^ concentration is averaged over short distances only (Figures 8D,E). Nevertheless, the error introduced by the 1D approach is much smaller than the errors caused by inaccurate morphologies (Figure 8C) and simulating 3D diffusion in tetrahedral meshes is quite slow. However, 3D diffusion must be included in biophysically accurate models of synaptic plasticity or models involving Ca^2+^ based signaling pathways.

For most of modeling projects with the goal of capturing excitability and integrative properties of dendrites, a correct implementation of 1D radial diffusion and buffering in NEURON (or any other compartment based simulator) will be an adequate approximation. It is then important to implement a variable number of submembrane shells, with larger number of shells in larger diameter compartments, and best using a fixed depth of the submembrane shell [DM_FD_ model: Figure 1, Equation (5)]. The model should be based on a high quality morphological reconstruction (Jacobs et al., 2010) and the variability of diameter along dendritic segments should be retained by having as many compartments as required to capture diameter changes (Figures 8D,E).

Finally, we do not recommend the use of simple pool models, unless good data on the properties of Ca^2+^ buffering (e.g., Schmidt et al., 2003 for PCs) in the neuron type to be modeled is completely absent. If one is forced to use a simple pool model, make sure it is implemented correctly [SP_new_, Figure 3A and Equations (2) and (6)].

### Conflict of interest statement

The authors declare that the research was conducted in the absence of any commercial or financial relationships that could be construed as a potential conflict of interest.
