# Supplementary material for: Dendritic diameters affect the spatial variability of intracellular calcium dynamics in computer models
Source: Front Cell Neurosci. 2014 Jul 23;8:168. doi: 10.3389/fncel.2014.00168 (PMC4107854; doi:10.3389/fncel.2014.00168)
Supplement: Supplementary file 1 [file DataSheet1.PDF]

Supplementary Information

Dendritic diameters affect the spatial variability of intracellular calcium dynamics in computer models

Haroon Anwar[1,2]*, Christopher J. Roome[3], Hermina Nedelescu[1,2]†, Weiliang Chen[2], Bernd Kuhn[3], Erik De Schutter[1,2]

[1]Theoretical Neurobiology and Neuroengineering, University of Antwerp, Wilrijk, Belgium
[2]Computational Neuroscience Unit, Okinawa Institute of Science and Technology, Onna-Son, Okinawa, Japan
[3]Optical Neuroimaging Unit, Okinawa Institute of Science and Technology, Onna-Son, Okinawa, Japan

*** Correspondence:** Haroon Anwar, Department of Biological Sciences,New Jersey Institute of Technology, University Heights, Newark, New Jersey 07102-1982, USA.
hanwar@njit.edu

† Brain Mechanisms for Behavior Unit, 1919-1 Tancha, Onna-Son, Kunigamo-gun, Okinawa 904-0495, Japan.

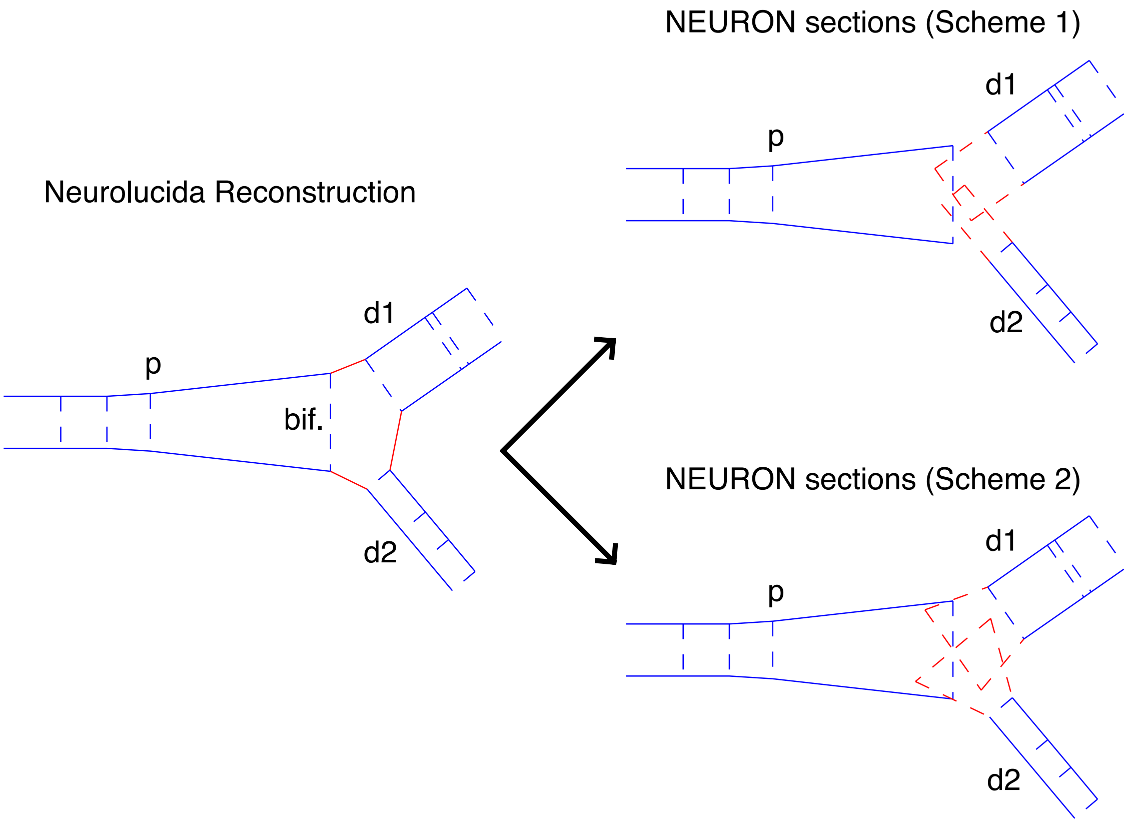

Figure S1: Conversion of reconstructed morphology into format suitable for NEURON simulation using CVapp changes diameters at branch points.

The dendritic diameters marked with blue broken lines in both left and right panels are reconstructed using Neurolucida. Parent dendritic segment is marked with ‘p’, child dendritic segments are marked with ‘d1’ and ‘d2’, and branching point is marked as ‘bif.’. The red solid line in left panel indicates untraced part. Conversion of morphology from ‘ASC’ file format to ‘HOC’ file format (as shown in Scheme 1 on top right panel) covers the untraced part by inserting the first traced diameter (marked with blue broken line) of each child segment as the first diameter of respective child dendritic segments (marked with red broken lines). Conversion of morphology from ‘SWC’ file format to ‘HOC’ file format (as shown in Scheme 2 on bottom right panel) covers the untraced part by inserting the traced diameter at the bifurcation (marked with blue broken line and ‘bif.’ in left panel) as first diameter of both child dendritic segments (marked with red broken lines).
